# Proactive, Recovery-Oriented Treatment Navigation to Engage Racially Diverse Veterans in Mental Healthcare (PARTNER-MH), a Peer-Led Patient Navigation Intervention for Racially and Ethnically Minoritized Veterans in Veterans Health Administration Mental Health Services: Protocol for a Mixed Methods Randomized Controlled Feasibility Study

**DOI:** 10.2196/37712

**Published:** 2022-09-06

**Authors:** Johanne Eliacin, Diana J Burgess, Angela L Rollins, Scott Patterson, Teresa Damush, Matthew J Bair, Michelle P Salyers, Michele Spoont, James E Slaven, Caitlin O'Connor, Kiara Walker, Denise S Zou, Emily Austin, John Akins, James Miller, Matthew Chinman, Marianne S Matthias

**Affiliations:** 1 Center for Health Information and Communication Health Services Research & Development Richard L Roudebush VA Medical Center Indianapolis, IN United States; 2 Regenstrief Institute Indianapolis, IN United States; 3 National Center for PTSD VA Boston Healthcare System Boston, MA United States; 4 Center for Care Delivery and Outcomes Research Minneapolis VA Healthcare System Minneapolis, MN United States; 5 Department of Medicine University of Minnesota Minneapolis, MN United States; 6 Department of Psychology Indiana University-Purdue University Indianapolis Indianapolis, IN United States; 7 Department of Psychiatry Richard L. Roudebush VA Medical Center Indianapolis, IN United States; 8 Department of Medicine Indiana University School of Medicine Indianapolis, IN United States; 9 Department of Biostatistics and Health Data Science Indiana University School of Medicine Indianapolis, IN United States; 10 Center for Health Equity Research and Promotion VA Pittsburgh Health Care Pittsburgh, PA United States; 11 RAND Corporation Pittsburgh Office Pittsburgh, PA United States

**Keywords:** Veterans, health care disparities, intervention, mental health, patient engagement, shared decision-making, patient navigation

## Abstract

**Background:**

Mental health care disparities are persistent and have increased in recent years. Compared with their White counterparts, members of racially and ethnically minoritized groups have less access to mental health care. Minoritized groups also have lower engagement in mental health treatment and are more likely to experience ineffective patient-provider communication, which contribute to negative mental health care experiences and poor mental health outcomes. Interventions that embrace recovery-oriented practices to support patient engagement and empower patients to participate in their mental health care and treatment decisions may help reduce mental health care disparities. Designed to achieve this goal, the Proactive, Recovery-Oriented Treatment Navigation to Engage Racially Diverse Veterans in Mental Healthcare (PARTNER-MH) is a peer-led patient navigation intervention that aims to engage minoritized patients in mental health treatment, support them to play a greater role in their care, and facilitate their participation in shared treatment decision-making.

**Objective:**

The primary aim of this study is to assess the feasibility and acceptability of PARTNER-MH delivered to patients over 6 months. The second aim is to evaluate the preliminary effects of PARTNER-MH on patient activation, patient engagement, and shared decision-making. The third aim is to examine patient-perceived barriers to and facilitators of engagement in PARTNER-MH as well as contextual factors that may inhibit or promote the integration, sustainability, and scalability of PARTNER-MH using the Consolidated Framework for Implementation Research.

**Methods:**

This pilot study evaluates the feasibility and acceptability of PARTNER-MH in a Veterans Health Administration (VHA) mental health setting using a mixed methods, randomized controlled trial study design. PARTNER-MH is tested under real-world conditions using certified VHA peer specialists (peers) selected through usual VHA hiring practices and assigned to the mental health service line. Peers provide PARTNER-MH and usual peer support services. The study compares the impact of PARTNER-MH versus a wait-list control group on patient activation, patient engagement, and shared decision-making as well as other patient-level outcomes. PARTNER-MH also examines organizational factors that could impact its future implementation in VHA settings.

**Results:**

Participants (N=50) were Veterans who were mostly male (n=31, 62%) and self-identified as non-Hispanic (n=44, 88%) and Black (n=35, 70%) with a median age of 45 to 54 years. Most had at least some college education, and 32% (16/50) had completed ≥4 years of college. Randomization produced comparable groups in terms of characteristics and outcome measures at baseline, except for sex.

**Conclusions:**

Rather than simply documenting health disparities among vulnerable populations, PARTNER-MH offers opportunities to evaluate a tailored, culturally sensitive, system-based intervention to improve patient engagement and patient-provider communication in mental health care for racially and ethnically minoritized individuals.

**Trial Registration:**

ClinicalTrials.gov NCT04515771; https://clinicaltrials.gov/ct2/show/NCT04515771

**International Registered Report Identifier (IRRID):**

DERR1-10.2196/37712

## Introduction

### Background

Low patient engagement in care and ineffective patient-provider communication are 2 major contributors to health care disparities [1-6]. Minoritized patients are less likely to be engaged in care, particularly in mental health care [5-7], which often leads to lower health service use [8-10], higher treatment dropout rates [5,11,12], and worse clinical outcomes [13]. Reasons for low engagement in mental health care vary but include perceived futility of treatment, inadequate access to care, lack of culturally sensitive treatment, low self-efficacy, and lack of trust in health care systems [14-16]. Minoritized patients are also more likely to experience poor patient-provider communication [1,17] and be excluded from treatment decisions [18]. Studies have found patient-provider interactions to be marked by conflicts, perceptions of discrimination, and provider dominance [18]. Ineffective patient-provider communication perpetuates racial health care disparities by contributing to poor care experiences [19-21], low treatment adherence [22], and negative health outcomes [23].

Recovery-oriented practices that prioritize person-centered care, patient autonomy, and empowerment may help reduce health care disparities by engaging patients in services and supporting them to play an active role in their care and treatment decisions [6,14]. Peer support specialists (peers) have been effective in engaging vulnerable populations at risk for treatment dropout, such as patients with serious mental illness, by serving as role models for patients in recovery, addressing stigma associated with mental illness, and providing support [24,25]. Moreover, health services interventions that are tailored to meet the unique needs of minoritized patients by providing culturally sensitive care and addressing patients’ social contexts may have a substantial impact [14,26]. One such intervention, patient navigation, is a well-established care model that is effective in reducing barriers to care for minoritized groups by providing personalized navigation services and addressing patients’ barriers to care [27-29].

### Addressing Mental Health Care Disparities: Piloting the Proactive, Recovery-Oriented Treatment Navigation to Engage Racially Diverse Veterans in Mental Healthcare Intervention

To maximize the potential benefits of a culturally sensitive and recovery-oriented approach to patient engagement and communication for minoritized groups, we developed a peer-led patient navigation program—Proactive, Recovery-Oriented Treatment Navigation to Engage Racially Diverse Veterans in Mental Healthcare (PARTNER-MH). This manuscript describes the study protocol for a randomized controlled trial to assess the feasibility and acceptability of PARTNER-MH and organizational factors that could impact its implementation in Veterans Health Administration (VHA) settings.

### PARTNER-MH Intervention

PARTNER-MH incorporates peer support and patient navigation care models to deliver a manualized patient activation, engagement, and communication intervention to racially and ethnically minoritized Veterans in VHA outpatient mental health clinics. The aims of PARTNER-MH are as follows: (1) to engage racially and ethnically minoritized patients in mental health care, (2) to increase patient activation by giving patients the tools to become active collaborators in their care, and (3) to improve patients’ communication skills and participation in shared treatment decision-making.

#### Area of Focus 1: Patient Activation and Patient Engagement

**Figure 1:**
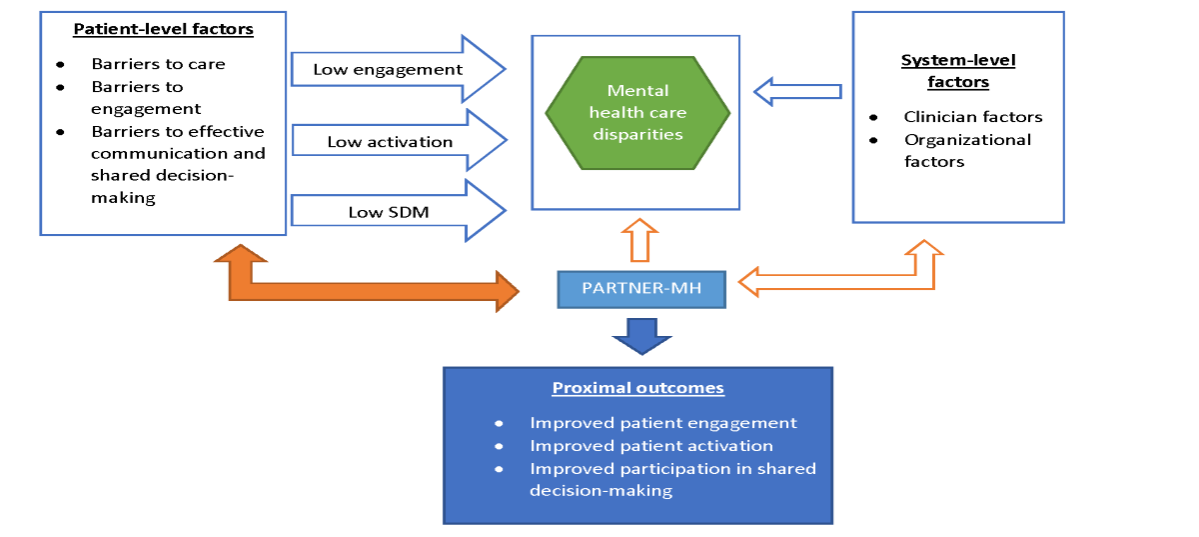
Proactive, Recovery-Oriented Treatment Navigation to Engage Racially Diverse Veterans in Mental Healthcare (PARTNER-MH) conceptual model. SDM: shared decision-making.

As depicted in Figure 1, PARTNER-MH is designed to reduce mental health disparities by activating and engaging racially and ethnically minoritized patients in VHA mental health services. Although patient activation is closely related to patient engagement and both are often used interchangeably in the literature, they are slightly distinct concepts [30]. Patient activation is an intrapsychic state or cognitive process that is a prerequisite for engagement in care. It is defined as understanding one’s role in the care process and having the knowledge, skills, and confidence to manage one’s health and health care [30,31]. Patient activation has been linked to positive health outcomes and health care experiences and decreased health care costs. Activated patients self-manage their health, collaborate with care providers, make decisions that affect their health and health care costs, and have the ability to navigate the health care system, obtain preventive care, and participate in proactive behaviors such as regular physical exercise to maintain their health [30]. Patient engagement is the behavioral manifestation of an activated person working in partnership with their care providers to improve their health care experiences and health outcomes. Patient engagement includes patient behaviors demonstrating active participation in care that are shaped by patient-provider relationships and the care environment [16]. In PARTNER-MH, patient engagement involves a continuous and evolving process that begins with treatment seeking, followed by various indicators of ongoing participation in direct care as well as organizational factors that address barriers to care, such as access to services.

To increase patient activation, PARTNER-MH uses a peer support care model to create early and ongoing relationships with patients, develop trust, and empower patients to take charge of their health and health care. PARTNER-MH peers provide education to raise awareness of available services, address patient-level barriers to care such as self-stigma or negative beliefs associated with mental illness and treatment, and provide individualized mental health treatment navigation services to assist with care coordination [6,24,25]. Peers also help patients understand their role in their care, how they can actively partner with care providers to manage their health, and how they can make the most of their participation in mental health services.

PARTNER-MH seeks to facilitate patient engagement by addressing patients’ social contexts such as their racial and social identities, living environment, and lived experiences that shape their health and health care experiences. The PARTNER-MH approach to patient engagement also involves addressing negative social determinants of health such as unmet social needs that might be preventing their engagement in care. These include food and housing insecurity, legal issues, and social isolation. Addressing patients’ unmet social needs also serves as a catalyst to engage them in conversations about what matters to them as well as social and cultural experiences that may affect their health and health care.

#### Area of Focus 2: Patient Communication

Another objective of PARTNER-MH is to improve patients’ communication with their providers by identifying and addressing barriers to effective patient-provider communication. This area of focus also includes helping patients prepare for their mental health visits by identifying goals, preparing questions for providers, supporting collaborative relationships with providers, and participating in shared treatment decision-making.

### Study Objectives

Aim 1 (primary aim) is to assess the feasibility and acceptability of PARTNER-MH in a VHA mental health care setting.

Aim 2 is to evaluate the preliminary effects of PARTNER-MH on patient activation, patient engagement, and shared decision-making (SDM). We hypothesize that patients randomized to the PARTNER-MH intervention group will report greater patient engagement, patient activation, and SDM than patients randomized to the control group.

Aim 3 is to examine patient-perceived barriers to and facilitators of engagement in PARTNER-MH as well as contextual factors, using the Consolidated Framework for Implementation Research (CFIR) [32], that may inhibit or promote the integration, sustainability, and scalability of PARTNER-MH.

## Methods

### Design Overview

This pilot study used a convergent mixed methods design [33] that involved a randomized controlled trial comparing the PARTNER-MH intervention with a wait-list control group with a sample of 50 racially and ethnically minoritized Veterans. The wait-list design was selected as a comparator for treatment as usual because it provides patients with the opportunity to participate in the intervention after the wait period, which facilitates recruitment into the study.

### PARTNER-MH Interventionists

The interventionists for this pilot study are 2 certified VHA peer support specialists, selected through usual VHA hiring practices and assigned to the mental health service line, who have completed the PARTNER-MH training program. The training program consists of 40 hours of didactic sessions that cover topics such as patient navigation, patient engagement, social determinants of health, diversity and racial discrimination in health care, effective communication, and professional development.

### Adherence to Intervention Protocol

Fidelity assessment was conducted quarterly using a sample of 8 patients in the active group (8/29, 28%) stratified by 2 peers. A total of 2 clinical psychologists from the study team used the PARTNER-MH fidelity 17-item checklist and audio-recorded intervention sessions or conducted live participant observations to assess fidelity. Fidelity assessment outcomes were then discussed with peers as well as the steps needed to reinforce or correct deviations from study procedures. In addition, peers receive weekly supervision to reinforce training information, address challenges, and provide support (aim 1).

### PARTNER-MH Development and Intervention Structure

PARTNER-MH is a theory-driven, peer-led intervention that was developed using participatory approaches [34,35] guided by the CFIR [32]. Specifically, this process involved the active participation of racially diverse Veterans, peers, and peer supervisors throughout the development and preimplementation phases of PARTNER-MH [36].

PARTNER-MH is a 6-month intervention that consists of individualized sessions with an assigned peer. Sessions are delivered in person, over the phone, or via videoconferencing, depending on patient preferences. Owing to the COVID-19 pandemic and restrictions on in-person visits, most of the sessions were delivered via telehealth. The PARTNER-MH sessions were delivered weekly for the first month, biweekly for the second and third months, and monthly thereafter. Peers and patients also met more often, as needed. The sessions lasted approximately an hour and were tailored to meet patient goals and needs related to engagement, access to services, care coordination, health care communication, and personal support. Peers used the PARTNER-MH handbook to guide and organize their sessions, but they also had the flexibility to use their lived experiences and training to inform the sessions. The flexibility of the PARTNER-MH structure also allowed patients to cover different modules at their own pace. Textbox 1 depicts the modules covered in the handbook and during the sessions.

**Proactive, Recovery-Oriented Treatment Navigation to Engage Racially Diverse Veterans in Mental Healthcare (PARTNER-MH) modules.**

**Getting to know you**

- This module covers topics related to social needs assessment (social determinants of health), rapport building, information about patients’ social contexts, strengths, racial and other identities, recovery story, and goal setting.

**Navigation to Veterans Health Administration mental health services**

- Information about Veterans Health Administration metal health services, treatment team composition, treatment options, and how to make the most of services are discussed in this module.

**Patient engagement**

- This module focuses on ways to be engaged in one’s care, discussions of engagement behaviors, and setting goals for being engaged.

**Planning your mental health visits**

- This module describes the importance of visit preparation, how to prepare for psychiatric medication and therapy visits, and how to set goals for visits that are aligned with one’s recovery goals.

**Effective patient-provider communication and shared decision-making**

- Shared decision-making and effective communication are discussed in this module. Patients role-play with peers and examine barriers to effective communication, strategies to improve communication, and collaboration with providers.

### Participants and Setting

PARTNER-MH was offered to racially and ethnically minoritized Veterans receiving mental health services from an outpatient mental health clinic at a large VHA medical center in the Midwest and associated community-based outpatient clinics. The program targeted Veterans across psychiatric diagnostic categories who were relatively new to the broad array but somewhat complicated configuration of VHA outpatient mental health clinics, often requiring help to navigate mental health services. To be eligible for the study, participants must (1) belong to a racially or ethnically minoritized group, (2) be aged ≥18 years, and (3) have a new medication management or therapy appointment scheduled within 12 months before enrollment in the study or have recently re-established treatment after an absence of 2 years. Veterans are excluded if they (1) have mental or cognitive impairments that limited their ability to give consent (eg, having acute psychotic symptoms or being cognitively impaired during the consent or interview process), (2) have hearing difficulties that prevent participation in the interviews, or (3) received medication management services at the clinic for >12 months before enrollment in the study.

### Recruitment

Participant recruitment for PARTNER-MH is complete. Multiple strategies were used to recruit participants to capture a diverse group of racially and ethnically minoritized patients. They included inviting eligible patients identified through electronic health records and sending them an introductory letter informing them about the study. The letter gave the participants a method for opting out of further contact. In the absence of such notification, 10 days after the letter’s receipt was expected, study staff called the patient to explain the study in greater detail, conduct initial screening, and ask eligible patients whether they wished to participate. Other recruitment strategies included clinician referrals, patient self-referrals, direct advertisements, and snowball sampling (ie, asking enrolled participants to refer others). All eligible patients were given a research packet that included an invitation letter and a study information sheet.

### Ethical Considerations

Approval was obtained from the Indiana University Institutional Review Board in November 2017 (1708628270) and the Veterans Affairs (VA) Research and Development review committee. Protocol modifications will undergo further review by the institutional review board, be communicated to the research team, and updated in the clinical trials registry.

### Randomization and Protection Against Sources of Bias

Participants completed baseline assessment before being randomized into the study arms to ensure balance and reduce selection bias. Allocation to the treatment arm was carried out using a computer-generated randomization list with randomly varying block sizes of 4 and 8 to maximize allocation concealment. Furthermore, although blinding was not feasible for this project because of the study’s limited staffing and the need to collect participant feedback on the feasibility and acceptability of the intervention, study personnel involved in screening and enrollment were masked to the computer-generated randomization assignment and were not included in delivering the intervention. Moreover, peers were not involved in data collection and did not have access to participants’ assessment results.

### Wait-list Control Structure and Overview

Participants in the wait-list control group received regular VHA mental health services (eg, individual or group psychotherapy, consults, and medication management) for the 6 months after enrollment. To overcome potential issues of contamination, where a peer could deliver PARTNER-MH services to control group participants, participants in the control group were encouraged not to use peer services unless they dropped out of the study. Chart reviews were conducted to assess contamination.

### Data Collection Methods, Data Management, and Monitoring

The data collection for this study is ongoing. Screening, enrollment, and survey data were collected and stored via VA REDCap (Research Electronic Data Capture; Vanderbilt University), behind the VA firewall. Outcomes were assessed over the phone at baseline and at 3, 6 (primary end point), 9, and 12 months. Outcome data also included qualitative interviews to evaluate participants’ experience of the intervention and organizational factors that may impact its future implementation and the integration of the quantitative and qualitative data. Study participants were compensated with a US $35 gift card for each assessment except for the primary end point (at 6 months), for which they received a US $50 gift card. In addition, because of the COVID-19 in-person visit restrictions, participants received a US $10 gift card for each month they remained enrolled in the study to facilitate access to telehealth delivery of the intervention and retention. A brief exit survey was sent to participants who discontinued the study to evaluate their experiences in the program. Participants’ enrollment in the study was recorded in their medical records, which peers had access to. A data safety and monitoring board was also established to evaluate the data quality and safety of the study.

### Aim 1 Outcomes and Analysis

Aim 1 is to assess the feasibility and acceptability of PARTNER-MH in a VHA mental health care setting.

The feasibility of PARTNER-MH will be determined based on participants’ recruitment, enrollment, and retention rates. Program acceptability for participants will be evaluated using session attendance, number of contacts with peer navigators, and the Patient Satisfaction Survey, which is an 11-item questionnaire rated on a 3-point Likert scale ranging from 1 (not at all) to 3 (very). Satisfaction with the peer was evaluated using a survey that included questions about the patient’s relationship with the peer and views of support provided by the peer. Descriptive summaries of recruitment, enrollment, retention, and satisfaction rates will be reported. Participant feedback from qualitative interviews will also be used to inform the feasibility and acceptability of PARTNER-MH (aim 3).

### Aim 2 Outcomes and Analysis

#### Overview

Aim 2 is to evaluate the preliminary effects of PARTNER-MH on patient engagement, patient activation, and SDM.

Aim 2 has three main outcome measures: patient activation, patient engagement, and SDM. In addition, sociodemographic data (eg, age, sex, race, ethnicity, education, and marital status) were collected at baseline. Tertiary and health-related outcomes that included communication self-efficacy, depression, mental health, and physical health functions were also assessed at all time points and are listed in the *Tertiary Outcomes* section.

#### Secondary Outcomes

The *Patient Activation Measure for Mental Health* (PAM-MH) [37] is a 13-item questionnaire that measures an individual’s perceived ability to manage illness and health behaviors. The PAM-MH is reliable, valid, and sensitive to change and correlates with measures of improved self-management and health outcomes. The questions are rated on a 4-point Likert-type scale and then converted using Rasch analysis to a 100-point scale. The PAM-MH has strong test-retest reliability and internal consistency (Cronbach α=.91).

Patient engagement will be assessed using the *Altarum Consumer Engagement (ACE)* measure, a 12-item measure that consists of 3 subscales to reflect patients’ commitment to everyday health behaviors, navigation skills in using health care services, and informed choice in treatment decisions [38]. The ACE is administered as a 5-level Likert scale. The subscale scores range from 5 to 25, and the total engagement score is computed by adding the 3 subscale scores and multiplying the sum by 4/3 to obtain a possible range score of 20 to 100. Higher scores represent higher patient engagement.

Finally, we will administer the *SDM-Q-9*, a widely used 9-item patient-reported SDM measure that focuses on the decisional process by rating providers’ behaviors in medical encounters. For this study, we will ask participants to think of their most recent visit with their mental health provider. The scale shows good internal consistency (α=.94) and high face and structural validity [39,40]. The SDM-Q-9 is rated on a 6-point Likert scale. The items are scored from 0 to 5 on a 6-point Likert scale ranging from “completely disagree” (0) to “completely agree” (5). A simple sum score with possible values between 0 and 45 is obtained. Item means range from 2.9 to 3.81, and the mean sum of SDM-Q-9 is 3.15 (SD 0.9) [41]. In addition to the SDM-Q-9, we added four questions to evaluate patient participation in treatment decision-making: (1) To the extent that SDM took place during your visit, how much did you drive the process? (2) Thinking about your goal for the visit (what you wanted to be done), how much do you feel you accomplished? (3) How much did you feel heard during your discussion with your provider? (4) Did you experience any barriers that kept you from speaking up or participating in SDM during that visit?

#### Tertiary Outcomes

*Loneliness* was assessed using the University of California Los Angeles Loneliness Scale Short Form, a 6-item scale with a 4-point Likert scale ranging from 1 (never) to 4 (often). It has demonstrated internal consistency (α=.89-.94) and test-retest reliability (*r*=0.73) [42].

The *Perceived Efficacy in Patient-Physician Interaction-5 (PEPPI-5) scale* measures patients’ self-efficacy in obtaining medical information and attention to their chief health concern from a physician [43]. The PEPPI-5 is 5-item scale scored on a 5-point Likert scale ranging from 1 (not confident at all) to 5 (very confident). Higher scores indicate higher levels of self-efficacy. The PEPPI-5 has internal consistency (α=.92) and adequate test-retest reliability.

The *Working Alliance Inventory*-*Short Revised* evaluates three key aspects of the therapeutic alliance between patients and their mental health providers: (1) agreement on the tasks of therapy, (2) agreement on the goals of therapy, and (3) development of an affective bond [44,45]. The *Working Alliance Inventory*-*Short Revised* includes 12 items rated on a 5-point Likert scale ranging from 1(never) to 5(always). It shows good psychometric properties in both outpatient and inpatient populations, with a reliability of Cronbach α>.90 and convergent validity with the helping alliance questionnaire (*r*>0.064).

The *Patient Health Questionnaire-9* measures the severity of depressive symptoms. The *Patient Health Questionnaire-9* includes 9 items and demonstrates high internal consistency and reliability (Cronbach α=.89) and good sensitivity and specificity for identifying cases of depression and assessing depression symptom severity [46].

The *Veterans RAND 12-item Health Survey* measures physical function, social function, role limitations owing to physical and emotional problems, mental health, energy and vitality, bodily pain, and the general perception of health. The *Veterans RAND 12-item Health Survey* uses 5-point ordinal response choices and provides two scores: the physical component summary score and the mental health summary score [47].

The *Perceived Discrimination in Healthcare Questionnaire* is a 7-item questionnaire that assesses a respondent’s overall health care experiences rather than a specific experience based on their racial background. Respondents are asked to rate their experiences on a 5-point Likert-type scale, with answers ranging from 0 (never) to 4 (always). This questionnaire has shown excellent reliability in diverse patient populations [48].

*Veterans’ trust in the VA* is assessed using a 3-item questionnaire. Responses range from “strongly disagree” to “strongly agree.”

#### Planned Statistical Analyses for Aim 2

Power calculations are provided, but as a pilot, this study is powered only to detect large differences between groups. With a sample of 22 participants in the intervention group and 15 in the wait-list control group, we have 80% power at a .05 significance level to detect an effect size of 0.965 for tests between groups using 2-sided 2-sample *t* tests. With an estimated SD of 14 for PAM-MH based on previous studies, this sample size will allow detection of a PAM-MH difference of 13.5 between the 2 groups. Within the intervention group, the study can detect an effect size of 0.626 for tests between time points using 2-sided paired *t* tests, for a difference of 8.8 for PAM-MH changes. Similarly, in the wait-list control group, the study can detect an effect size of 0.778 and a difference of 10.8 for PAM-MH changes. To account for 25% attrition during follow-up, the study enrolled 30 intervention participants and 20 wait-list control participants.

The internal consistency of each scale for primary and secondary outcomes will be verified in this study sample using Cronbach α. Distributions of the scale scores will be examined to determine whether transformation of the data or nonparametric tests are required for the analyses. In this study, 2-sample *t* tests and Fisher exact tests for continuous and categorical variables, respectively, will be used to compare the demographic and baseline data between participants with and without complete data. Repeated measures ANOVA (RMANOVA) for the scale scores will be used to compare data among the assessments over time. The RMANOVAs will allow different correlations between each assessment time and will allow for the appropriate covariance structure to model the intraparticipant correlations; they will also include a random effect for peers to account for correlation among participants with the same peer. In this intent-to-treat analysis, the RMANOVAs will provide unbiased estimates under the missing-at-random assumption. A 5% significance level will be used for each test.

#### Planned Mixed Methods Analysis for Aim 2

**Figure 2:**
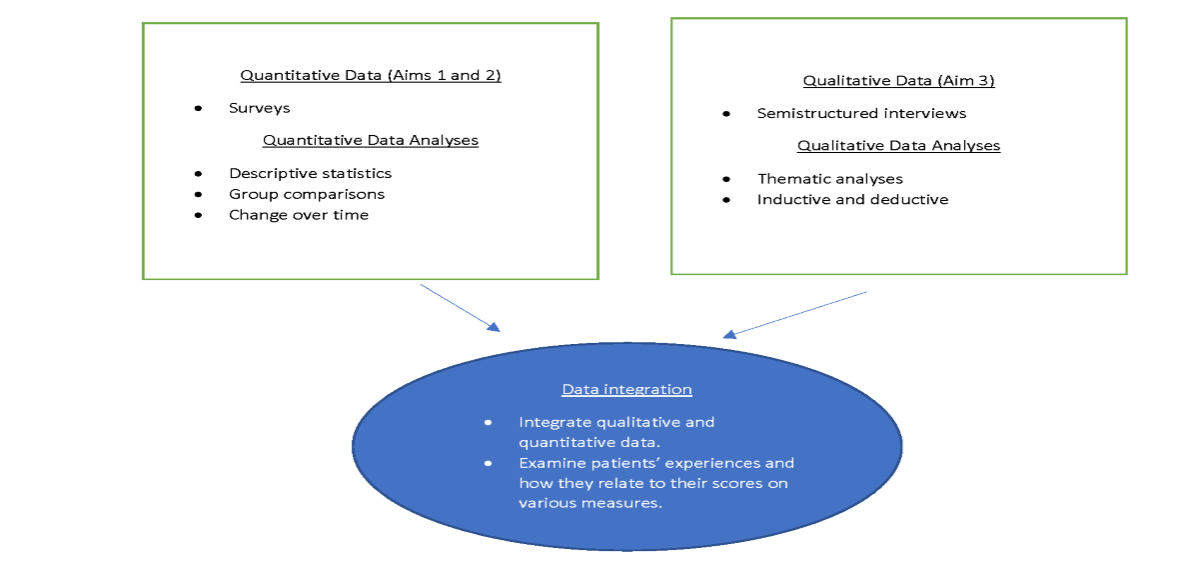
Joint display for mixed methods data collection and analysis.

As depicted in Figure 2, this study uses a convergent mixed methods design [33], which involves simultaneously collecting quantitative and qualitative data and giving equal weight to these data in analyses for the purposes of gaining breadth and depth of understanding (ie, complementarity), identifying whether the qualitative and quantitative data provide the same answer to the same question (ie, convergence), and using qualitative data to expand on unexpected quantitative findings (explanatory) [49-51]. Planned mixed data analysis will involve merging and comparing quantitative and qualitative data in parallel to interpret and explain the findings (QUAL+QUAN). This approach will enable us to triangulate our data by incorporating themes from the semistructured interviews and results from the self-report measures to validate our findings, especially in the context of this feasibility study. Many of the constructs assessed in the quantitative measures will also be explored in the qualitative interviews, for example, intervention characteristics (patient engagement, patient activation, and communication). Moreover, we will use an explanatory mixed methods approach consistent with a randomized controlled trial to better understand the quantitative findings, the process of the intervention, and participants’ experiences. The qualitative data will enhance the quantitative analyses by laying the groundwork to better understand the mechanisms of the intervention and facilitate its future implementation.

### Aim 3 Outcomes and Analysis

#### Overview

Aim 3 is to examine patient-perceived barriers to and facilitators of engagement in PARTNER-MH, as well as contextual factors that may inhibit or promote the integration, sustainability, and scalability of PARTNER-MH using the CFIR [32].

We will use domains of the CFIR [32] to collect and analyze data to inform aim 3. The CFIR offers an overarching typology of five domains affecting intervention development and implementation: (1) intervention characteristics, (2) inner setting, (3) outer setting, (4) characteristics of individuals, and (5) implementation process [32]. Briefly, intervention characteristics include evidence of the intervention and its adaptability. Implementation takes place within an inner setting—the program providing the service. The inner setting is affected by the outer setting—the broader treatment system. Characteristics of individuals such as their skills level also affect intervention delivery and implementation. The implementation process involves different strategies and tools that are used for putting a new practice in place.

Data collection for aim 3 will be guided by the CFIR using semistructured interviews. We will conduct interviews with patients and providers to obtain their perspectives on the intervention and on their experiences of participating in the PARTNER-MH program. Interviews will also assess organizational factors such as time and other resources that may affect the delivery and content of the intervention as well as the impact of the program on Veteran outcomes.

#### Aim 3 Study Participants

We will invite all 30 Veterans from the intervention group to participate in a qualitative interview. In addition, we will include a purposeful sample of 5 mental health staff members (prescribing and nonprescribing clinicians) with experience in working with peers and patients enrolled in the program.

#### Aim 3 Planned Qualitative Data Analysis

Interviews from aim 3 will be transcribed, deidentified, and entered into NVivo (QSR International), a qualitative analytical software program, to help organize the data. To facilitate the completion of qualitative data coding and analysis in a short time frame, we will incorporate several features recommended in rapid qualitative assessment [52]. First, we will impose some structure on the data being analyzed. The interviews will reflect CFIR constructs, which will allow for easier access to apply coding. Second, we will incorporate selected codes a priori, based on our prior research, to provide initial structure to the coding process, but will also allow for the expansion of the code list in which other meaningful ideas may emerge. We will use an inductive, interpretive approach that borrows concepts from grounded theory, to identify and explore emerging areas not covered by interview guidelines.

Through an iterative, consensus-building process, we will review transcripts to identify emergent themes consistent with techniques of immersion and crystallization [50]. We will independently read a few selected documents to identify possible areas of pursuit. We will create episode profiles for each transcript to facilitate in-depth understanding of each case and identify emerging themes for cross-transcript comparison. We will meet to discuss our findings and develop a working set of codes to add to the structural codes mentioned earlier. We will repeat this process on fresh sets of documents until we have a set of defined codes that are stable and consistent. We will then code individual transcripts independently.

To facilitate the rigor of the data analysis process, we plan to hold regular meetings with the coding team to examine coding across analysts, resolve differences in coding, identify and resolve coding drift, and conduct iterative refinement of code definitions. We will maintain memos of our coding processes, coding decisions, and analyses. We will also continually assess and maintain consistency and consensus in our coding practices [50].

## Results

### Overview

**Figure 3:**
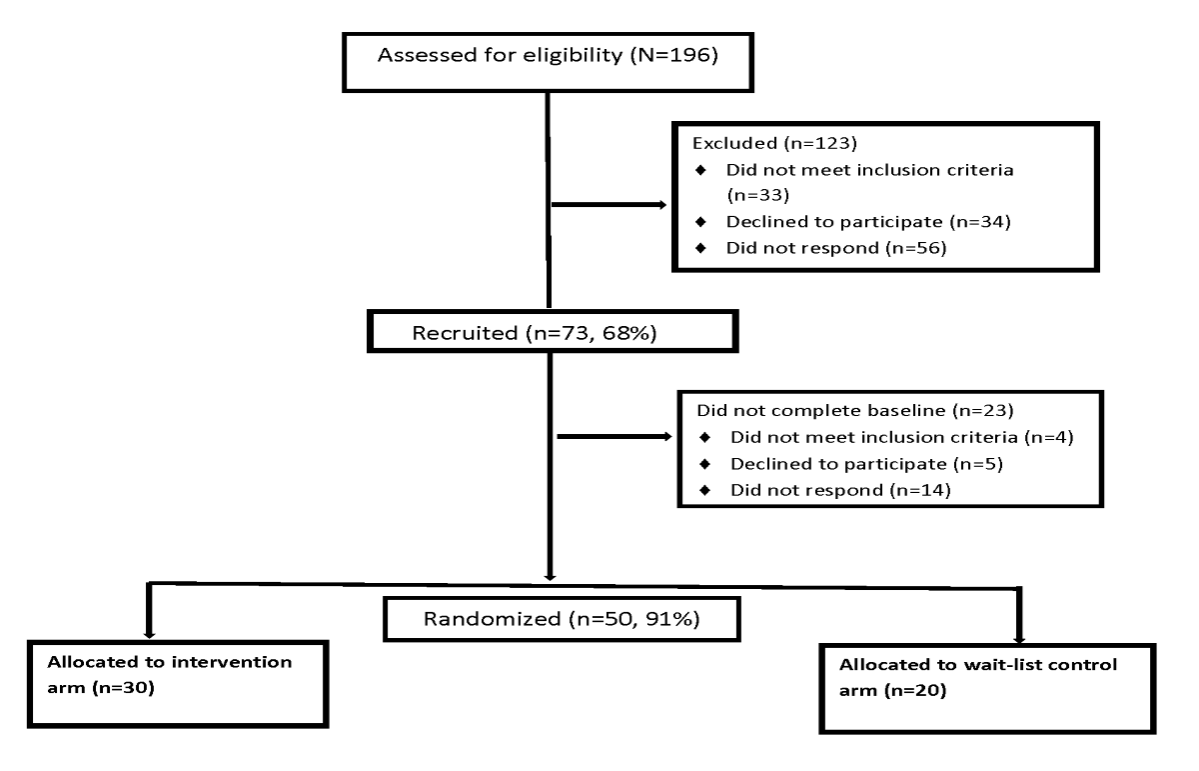
Proactive, Recovery-Oriented Treatment Navigation to Engage Racially Diverse Veterans in Mental Healthcare CONSORT (Consolidated Standards of Reporting Trials) flow diagram.

Figure 3 shows the results of the screening, eligibility determination, enrollment, and randomization of participants, conducted from August 17, 2020, to April 12, 2021. To recruit participants, we mailed letters to 191 potential participants. In addition, 5 patients were referred by clinicians or self-referred through study advertisements or word of mouth. Of these participants, 56 (29%) were not able to be reached to screen for eligibility, 33 (17%) were found to be ineligible, and 34 (17%) declined to participate. Of the interested participants, 73 (68%) met the eligibility criteria. However, 14 (19%) of these participants either canceled or did not show up for their baseline after rescheduling, 4 (5%) declined to participate in the study, and 4 (5%) were deemed ineligible because of changed circumstances such as relocating to a different state or transferring health services outside the VHA. Overall, 50 participants were enrolled in the study, with 30 (60%) randomized to the PARTNER-MH group and 20 (40%) randomized to the wait-list control group.

### Baseline Data

Participant demographics are presented in Table 1. The participants were mostly male (31/50, 62%) and self-identified as Black (35/50, 70%) and non-Hispanic (44/50, 88%). Participants were almost evenly distributed across three age groups: 25- to 34 years (12/50, 25%), 45 to 54 years (14/50, 28%), and 55 to 64 years (12/50, 24%), and 48% (24/50) had some college education. Randomization produced comparable groups with regard to baseline characteristics of age, race, ethnicity, and education. However, the wait-list control group had more female participants (11/20, 55%) than the active intervention group (8/30, 27%; *P*=.04). As shown in Tables 2 and 3, the 2 groups were also comparable on the outcome measures at baseline.

**Table 1:** Participant demographics.

Demographics			Overall (N=50), n (%)		Intervention group (n=30), n (%)		Wait-list control group (n=20), n (%)		*P* value	
**Age (years)**										.31
	18-24	1 (2)		0 (0)		1 (5)				
	25-34	12 (24)		8 (26.7)		4 (20)				
	35-44	9 (18)		7 (23.3)		2 (10)				
	45-54	14 (28)		6 (20)		8 (40)				
	55-64	12 (24)		7 (23.3)		5 (25)				
	65-75	2 (4)		2 (6.7)		0 (0)				
**Race**										.21
	White	3 (6)		3 (10)		0 (0)				
	Black	35 (70)		20 (66.7)		15 (75)				
	Asian	1 (2)		0 (0)		1 (5)				
	Other	5 (10)		2 (6.7)		3 (15)				
	Multi	6 (12)		5 (16.7)		1 (5)				
	Hispanic	6 (12)		4 (13.3)		2 (10)		.72		
**Sex**										.04^a^
	Male	31 (62)		22 (73.3)		9 (45)				
	Female	19 (38)		8 (26.7)		11 (55)				
**Education**										.07
	HS^b^ or GED^c^	10 (20)		3 (10)		7 (35)				
	Some college or 2 year degree	24 (48)		17 (56.7)		7 (35)				
	4-year college degree	9 (18)		7 (23.3)		2 (10)				
	>4 years college	7 (14)		3 (10)		4 (20)				

^a^Statistically significant.

^b^HS: high school.

^c^GED: General Educational Development.

**Table 2:** Baseline secondary outcome measures.

Measures			Overall (N=50)		Intervention group (n=30)		Wait-list control group (n=20)					*P* value (95% CI)
Altarum Consumer Engagement Commitment to Everyday Health Behavior subscale, mean (SD)			13.7 (5.2)		13.7 (4.7)		13.6 (5.9)					.91
Altarum Consumer Engagement Informed Choice subscale, mean (SD)			10.5 (5.0)		10.6 (4.8)		10.4 (5.4)					.89
Altarum Consumer Engagement Navigation subscale, mean (SD)			15.2 (3.8)		15.5 (4.4)		14.8 (2.8)					.41
Patient Activation Measure for Mental Health Activation scores, mean (SD)			51.5 (11.3)		52.8 (11.0)		49.6 (11.8)					.38
SDM-Q-9^a^, mean (SD)			26.8 (9.4)		25.8 (9.1)		28.3 (9.7)					.23
**SDM-Q-9 question 10, n (%)**											.86	
	Not at all	10 (20)		6 (20)		4 (20)						
	A little	10 (20)		6 (20)		4 (20)						
	Some	9 (18)		4 (13)		5 (25)						
	A lot	15 (30)		10 (33)		5 (25)						
	N/A^b^	6 (12)		4 (1)		2 (10)						
**SDM-Q-9 question 11, n (%)**											.91	
	Nothing at all	7 (14)		5 (16.7)		2 (10)						
	A little	16 (32)		9 (30)		7 (35)						
	Some	12 (24)		7 (23.3)		5 (25)						
	A lot	11 (22)		6 (20)		5 (25)						
	Every goal set	4 (8)		3 (10)		1 (5)						
**SDM-Q-9 question 12, n (%)**									.06			
	Not at all	4 (8)		4 (13.3)		0 (0)						
	A little	4 (8)		0 (0)		4 (20)						
	Some	9 (18)		5 (16.7)		4 (20)						
	A lot	22 (44)		14 (46.7)		8 (40)						
	Completely	11 (22.2)		7 (23.3)		4 (20)						
**SDM-Q-9 question 13, n (%)**									.97			
	Yes	16 (32)		10 (33.3)		6 (30)						
	No	29 (58)		17 (56.7)		12 (60)						
	Unsure	5 (10)		3 (10)		2 (10)						

^a^SDM-Q-9: shared decision-making-9.

^b^N/A: not applicable.

**Table 3:** Baseline tertiary outcome measures.

Secondary measures	Overall (N=50), mean, (SD)	Intervention group (n=30), mean, (SD)	Wait-list control group (n=20), mean, (SD)	*P* value
University of California, Los Angeles Loneliness Scale	16.2 (5.2)	17.1 (4.2)	14.8 (6.3)	.22
Perceived Efficacy in Patient-Physicians Interaction-5	35.7 (10.8)	35.2 (10.6)	36.4 (11.4)	.61
Working Alliance Inventory-Short Revised	39.8 (14.2)	38.0 (15.4)	42.6 (11.9)	.39
Perceived Discrimination in Healthcare Questionnaire	7.3 (5.8)	7.9 (5.9)	6.4 (5.7)	.30
Patient Health Questionnaire-9	13.6 (6.5)	14.9 (6.4)	11.5 (6.3)	.05
Veterans RAND 12-item Health Survey Physical Health	41.6 (7.7)	41.7 (7.2)	41.5 (8.8)	.98
Veterans RAND 12-item Health Survey Mental Health	32.4 (8.2)	31.0 (9.2)	34.7 (5.8)	.06

### Data Collection

Data collection for the trial ended in May 2022. Data analysis is projected to be completed by December 1, 2022.

## Discussion

### Overview

This pilot study aims to evaluate the feasibility, acceptability, and preliminary effects of a peer-led patient navigation intervention for racially and ethnically minoritized Veterans in VHA mental health clinics. We anticipate that the findings of this study will help identify barriers to and facilitators of the delivery of the intervention, its feasibility in VHA clinical settings, and its acceptability to the study participants. This pilot study will also facilitate the evaluation of the preliminary impacts of the intervention on patient engagement, patient activation, SDM, and related health outcomes.

Mental health care disparities are persistent and contribute to increased comorbidities, mortality, and health care cost expenditures among individuals of racially and ethnically minoritized backgrounds [53-55]. Minoritized patients experience lower activation, lower engagement, and lower participation in SDM, all of which have been implicated in negative health care experiences and health care outcomes for these groups [7,56,57]. Improving patient engagement, activation, or participation in SDM may lead to improved mental health outcomes and mental health equity in minoritized groups.

To move beyond the documentation of disparities, PARTNER-MH was designed to leverage the potential of peer support and patient navigation care models to effectively improve patient engagement, activation, and participation in SDM in mental health care among patients of minoritized backgrounds. PARTNER-MH uses a social determinant health care framework by assessing patients’ unmet social needs to engage them in care and learn about their lived experiences and social contexts. The additional focus of PARTNER-MH on improving patients’ communication self-efficacy and participation in SDM may contribute to improved satisfaction with services, treatment adherence, and outcomes. The program’s delivery over 6 months may also increase the percentage of patients who become engaged in care and achieve their mental health goals.

This feasibility trial will also help identify potential unanticipated challenges in the program and its implementation. For instance, it may help identify patients who may benefit the most from this intervention and the optimal length of intervention to facilitate sustained engagement in the program. It may also help identify interventionist characteristics and setting contexts that are most appropriate for this intervention.

### Limitations

A limitation of this study is that participants were not blinded to the study conditions, which may have affected participant behaviors and study outcomes. Moreover, PARTNER-MH is a patient-facing intervention, which may limit its impact on provider behaviors and ultimately, patient participation in collaborative treatment decision-making with their providers. Although this is a potential limitation of the intervention, other studies have shown that patient-focused interventions have some success in improving patient-provider communication and reducing health care disparities. For example, a patient-coaching intervention was shown to reduce patient-provider miscommunication and disparities in pain control among minoritized patients [58]. By supporting patients’ active engagement in care and fostering communication self-efficacy, PARTNER-MH may contribute to patients’ increased engagement in shared treatment decision-making. PARTNER-MH represents a novel approach that may help advance health equity for minoritized patients and represent a new system-based model to create sustained engagement of minoritized groups in mental health care.

In addition, as PARTNER-MH focuses on patient engagement, activation, and patient-provider communication—issues that are cross-cutting among other disease populations—the lessons learned in this study could be applied to minoritized patients with other chronic health conditions in other settings. PARTNER-MH also offers the potential to advance the field of peer support and patient navigation by creating a training program for VHA peer support specialists to deliver peer-led navigation services in outpatient mental health clinics over 6 months.

### Strengths of This Study

The mixed methods approach is a strength of this study that will help evaluate participants’ experiences of the intervention and identify areas of improvement and contextual factors that could influence its future implementation. In addition, the feasibility of PARTNER-MH is tested under real-world conditions such as using existing VHA hiring procedures and assigning study peers to the mental health organizational chart with mixed roles to provide PARTNER-MH and usual peer support services. This aspect of the study approach is also a strength that should provide rich implementation information for future consideration.

### Future Directions

This pilot study will lay the foundation for future testing of PARTNER-MH and contribute to mental health disparities intervention research that targets underrepresented, minoritized patients. The proposed study will provide preliminary data for a larger trial to examine the effectiveness of PARTNER-MH. On the basis of the findings of this pilot study, future studies may also address a broader array of clinical and health services outcomes such as the impact of PARTNER-MH on patients’ use of mental health services and treatment outcomes. They may also identify potential implementation strategies for PARTNER-MH and evaluate its economic impact on the health care expenditure of the VHA. Future trials are also needed to determine the broad-based acceptance and effectiveness of PARTNER-MH across diverse VA facilities.

### Dissemination Plan

The results of this study will be made available to health care professionals, researchers, and the public through publications, academic conferences, and other presentations. Study results will also be presented to VHA patient engagement boards, clinical partners, and other stakeholders.

### Conclusions

The outcome of this study will establish the feasibility and acceptability of PARTNER-MH, a peer-led patient navigation intervention to improve patient engagement, patient activation, and participation in SDM among racially diverse Veterans in mental health clinics. If the findings of this pilot study are positive, they will provide support for rigorous testing of PARTNER-MH in a larger trial. If found to be effective then, PARTNER-MH will significantly affect the mental health care experiences and outcomes of racially diverse patient populations. Moreover, as a peer-led intervention, PARTNER-MH could be promoted as a potentially easily scalable approach to increase mental health equity.

## Abbreviations

ACE: Altarum Consumer Engagement
CFIR: Consolidated Framework for Implementation Research
PAM-MH: Patient Activation Measure for Mental Health
PARTNER-MH: Proactive, Recovery-Oriented Treatment Navigation to Engage Racially Diverse Veterans in Mental Healthcare
PEPPI-5: Perceived Efficacy in Patient-Physician Interaction-5
REDCap: Research Electronic Data Capture; Vanderbilt University
RMANOVA: repeated measures ANOVA
SDM: shared decision-making
VA: Veterans Affairs
VHA: Veterans Health Administration

## Data Availability

Data are not available to share due to Department of Veterans Affairs and ethical retractions; however, annotation instructions and vocabulary are available upon request.
